# Supplementary material for: The use of virtual exhibition to promote exhibitors’ pro-environmental behavior: The case study of Zhejiang Yiwu International Intelligent Manufacturing Equipment Expo
Source: PLoS One. 2023 Nov 16;18(11):e0294502. doi: 10.1371/journal.pone.0294502 (PMC10653458; doi:10.1371/journal.pone.0294502)
Supplement: S1 File — (DOCX) [file pone.0294502.s002.docx]

**Appendix 1. The questionnaire of this study**

Dear Sir (Madam):

Thank you very much for taking time to participate in this project. This is an academic questionnaire survey. The purpose is to understand your/your company’s experience regarding to this 2021 Zhejiang Yiwu International Intelligent Manufacturing Equipment Expo. This questionnaire is anonymous, and the results are for academic research purposes only. Please feel free to fill it out. Thanks for your assistance!

**1. Screen question:**

“Do you know the main theme of this virtual exhibition is “green” that focuses on environmental protection?”

□Yes □No

Section 1: virtual exhibition experience

The following statements (**1~21)** are about the experience in 2021 Zhejiang Yiwu International Intelligent Manufacturing Equipment Expo. Please rate your **level of agreement** to each of the statements by choosing from the numbers 1~7 (1-**Strongly Disagree**, 7-**Strongly Agree**).

|  | **Strongly Disagree** | **Disagree** | **Somewhat Disagree** | **Neutral** | **Somewhat Agree** | **Agree** | **Strongly Agree** |
| --- | --- | --- | --- | --- | --- | --- | --- |
| 1. I was in control of my movement through the virtual environment (website). | □1 | □2 | □3 | □4 | □5 | □6 | □7 |
| 1. I had some control over the landscapes I wanted to see in the virtual experience. | □1 | □2 | □3 | □4 | □5 | □6 | □7 |
| 1. I was in control over my location. | □1 | □2 | □3 | □4 | □5 | □6 | □7 |
| 1. The virtual tourism technology responded to my needs quickly and efficiently. | □1 | □2 | □3 | □4 | □5 | □6 | □7 |
|  | | | | | | | |
| 1. I can join a virtual tour at any time. | □1 | □2 | □3 | □4 | □5 | □6 | □7 |
| 1. I can join a virtual tour from anywhere. | □1 | □2 | □3 | □4 | □5 | □6 | □7 |
| 1. I can participate in a virtual tour when I need to | □1 | □2 | □3 | □4 | □5 | □6 | □7 |
|  | | | | | | | |
| 1. I thought the sensory information provided by the screen was highly vivid. | □1 | □2 | □3 | □4 | □5 | □6 | □7 |
| 1. I thought the sensory information provided by the screen was highly rich. | □1 | □2 | □3 | □4 | □5 | □6 | □7 |
| 1. I thought the sensory contents provided by the screen was highly detailed. | □1 | □2 | □3 | □4 | □5 | □6 | □7 |
|  | | | | | | | |
| 1. The view of the museum in the VR tour is harmonious | □1 | □2 | □3 | □4 | □5 | □6 | □7 |
| 1. The museum environment as seen through the VR tour is quite attractive | □1 | □2 | □3 | □4 | □5 | □6 | □7 |
| 1. The museum as seen in the VR tour is quite visually appealing. | □1 | □2 | □3 | □4 | □5 | □6 | □7 |
| 1. The museum view as seen through the VR tour provided a way for users to easily experience it. | □1 | □2 | □3 | □4 | □5 | □6 | □7 |
|  | | | | | | | |
| 1. The virtual platform is effective in gathering consumers’ feedback. | □1 | □2 | □3 | □4 | □5 | □6 | □7 |
| 1. The virtual platform gives consumers the opportunity to talk back. | □1 | □2 | □3 | □4 | □5 | □6 | □7 |
| 1. The virtual platform facilitates interactive communication between consumers and knowledge contributors. | □1 | □2 | □3 | □4 | □5 | □6 | □7 |
| 1. The virtual platform facilitates interactive communication among consumers. | □1 | □2 | □3 | □4 | □5 | □6 | □7 |
|  | | | | | | | |
| 19. The choice of this virtual exhibition due to its environmental commitment makes me happy | □1 | □2 | □3 | □4 | □5 | □6 | □7 |
| 20. I consider it is correct to stay in this virtual exhibition because of its environmental commitment | □1 | □2 | □3 | □4 | □5 | □6 | □7 |
| 21. I am satisfied with this virtual exhibition because of its environmental performance. | □1 | □2 | □3 | □4 | □5 | □6 | □7 |
|  | | | | | | | |
| 22. The virtual exhibition efforts have significantly reduced waste within the production process. | □1 | □2 | □3 | □4 | □5 | □6 | □7 |
| 23. The virtual exhibition efforts have significantly improved product quality. | □1 | □2 | □3 | □4 | □5 | □6 | □7 |
| 24. Focusing on virtual exhibition has enhanced our facility’s reputation. | □1 | □2 | □3 | □4 | □5 | □6 | □7 |
| 25. The virtual exhibition efforts have led to improved facility performance. | □1 | □2 | □3 | □4 | □5 | □6 | □7 |
|  | | | | | | | |
| 26. Our corporation is willing to adopt eco-exhibiting. | □1 | □2 | □3 | □4 | □5 | □6 | □7 |
| 27. Corporations similar to ours are willing to adopt eco-exhibiting. | □1 | □2 | □3 | □4 | □5 | □6 | □7 |
| 28. Our corporation is willing to adopt eco-exhibiting during the next 3 years. | □1 | □2 | □3 | □4 | □5 | □6 | □7 |

Section 2: Demographic profile

The following questions (**1~3)** are to collect your personal information. Please be relieved that the information will be well protected and be used in this research only.

1. Your gender:

□Male □Female

1. Your company’s size:

□1-50 □51-100 □101-150 □151-200 □over 200

3. The past experiences of online/virtual exhibition:

□First time □Second time □Three times and above

The end of the questionnaire!

Thanks for your assistant!
